# High-Density Lipoprotein Cholesterol as a Potential Medium between Depletion of *Lachnospiraceae* Genera and Hypertension under a High-Calorie Diet

**DOI:** 10.1128/spectrum.02349-22

**Published:** 2022-10-17

**Authors:** Yongmei Lan, Kang Ning, Yanqing Ma, Jin Zhao, Caihong Ci, Xiao Yang, Fulong An, Zilong Zhang, Yan An, Mingyue Cheng

**Affiliations:** a Key Laboratory of Environmental Ecology and Population Health in Northwest Minority Areas, Department of Medicine, Northwest Minzu University, Lanzhou, China; b Key Laboratory of Molecular Biophysics of the Ministry of Education, Hubei Key Laboratory of Bioinformatics and Molecular Imaging, Center of AI Biology, Department of Bioinformatics and Systems Biology, College of Life Science and Technology, Huazhong University of Science and Technology, Wuhan, China; c Department of Internal Medicine, Department of Surgery, People’s Hospital of Sunan County, Zhangye, China; University of Nevada—Reno

**Keywords:** gut microbiota, hypertension, high-calorie diet, ethnic group, *Lachnospiraceae*, butyrate, high-density lipoprotein cholesterol, systolic blood pressure

## Abstract

Gut microbial dysbiosis has been associated with hypertension. An extremely high incidence of essential hypertension was found in the Han and the Yugur people who resided in Sunan County in China’s nomadic steppes, with little population movement. To investigate gut microbial contributions to this high incidence of hypertension, we recruited a total of 1, 242 Yugur and Han people, who had resided in Sunan County for more than 15 years and accounted for 3% of the local population. The epidemiological survey of 1,089 individuals indicated their nearly 1.8-times-higher prevalence of hypertension (38.2 to 43.3%) than the average in China (23.2%), under a special high-calorie diet based on wheat, cattle, mutton, and animal offal. Investigations of the fecal microbiota of another cohort of 153 individuals revealed that certain *Lachnospiraceae* genera were positively correlated with high-density lipoprotein cholesterol (HDL-C) but negatively correlated with systolic blood pressure (SBP) and diastolic blood pressure (DBP). HDL-C was negatively correlated with SBP and DBP. We further observed that the serum butyrate content was lower in both Han and Yugur people with hypertension than in those without hypertension. This study gives novel insight into the role of gut microbial dysbiosis in hypertension modulation under a high-calorie diet, where the notable depletion of *Lachnospiraceae* genera might lead to less production of butyrate, contributing to the lower level of HDL-C and elevating blood pressure in hypertension.

**IMPORTANCE** Dietary nutrients can be converted by the gut microbiota into metabolites such as short-chain fatty acids, which may serve as disease-preventing agents in hypertension. Due to the limited population mobility and unique high-calorie dietary habits, the cohort of this study can serve as a representative cohort for elucidating the associations between the gut microbiota and hypertension under a high-calorie diet. Moreover, low levels of HDL-C have previously been associated with an increased risk of various cardiovascular diseases (CVDs). Our findings provide new insight showing that low levels of HDL-C may be a potential medium between the depletion of *Lachnospiraceae* genera and hypertension under a high-calorie diet, which might also be a potential candidate for other CVDs.

## INTRODUCTION

The human gut microbiota has been correlated with the pathogenesis of a variety of cardiovascular diseases (CVDs), such as hypertension (1, 2). Hypertension is a major modifiable risk factor for CVDs such as myocardial infarction, heart failure, and stroke (3, 4). Compared to genetic effects, which contribute less than 20% to the risk of developing CVD pathogenesis, environmental effects, especially diet, are known for their prominent role in CVD pathogenesis (1, 5–7). Additionally, a diet rich in fruits, vegetables, and low-fat dairy products with reduced saturated and total fat has been confirmed to ameliorate hypertension in multiple randomized controlled trials (8). Moreover, the gut microbiota, whose composition is dominantly modulated by diet (9, 10), can convert dietary nutrients into metabolites such as short-chain fatty acids (SCFAs), which act as the potential disease-preventing factors in hypertension (1, 11). Indeed, our epidemiology survey showed that local Yugur and Han people, who resided in Sunan County in East Asia’s nomadic steppes, with little population movement, followed high-calorie dietary customs and presented an extremely high incidence of essential hypertension (see Table S1 in the supplemental material). Here, we have investigated the gut microbiota of local Han and Yugur people, with or without essential hypertension, to gain insight into the potential microbial contribution to their high incidence of hypertension.

**FIG 1:**
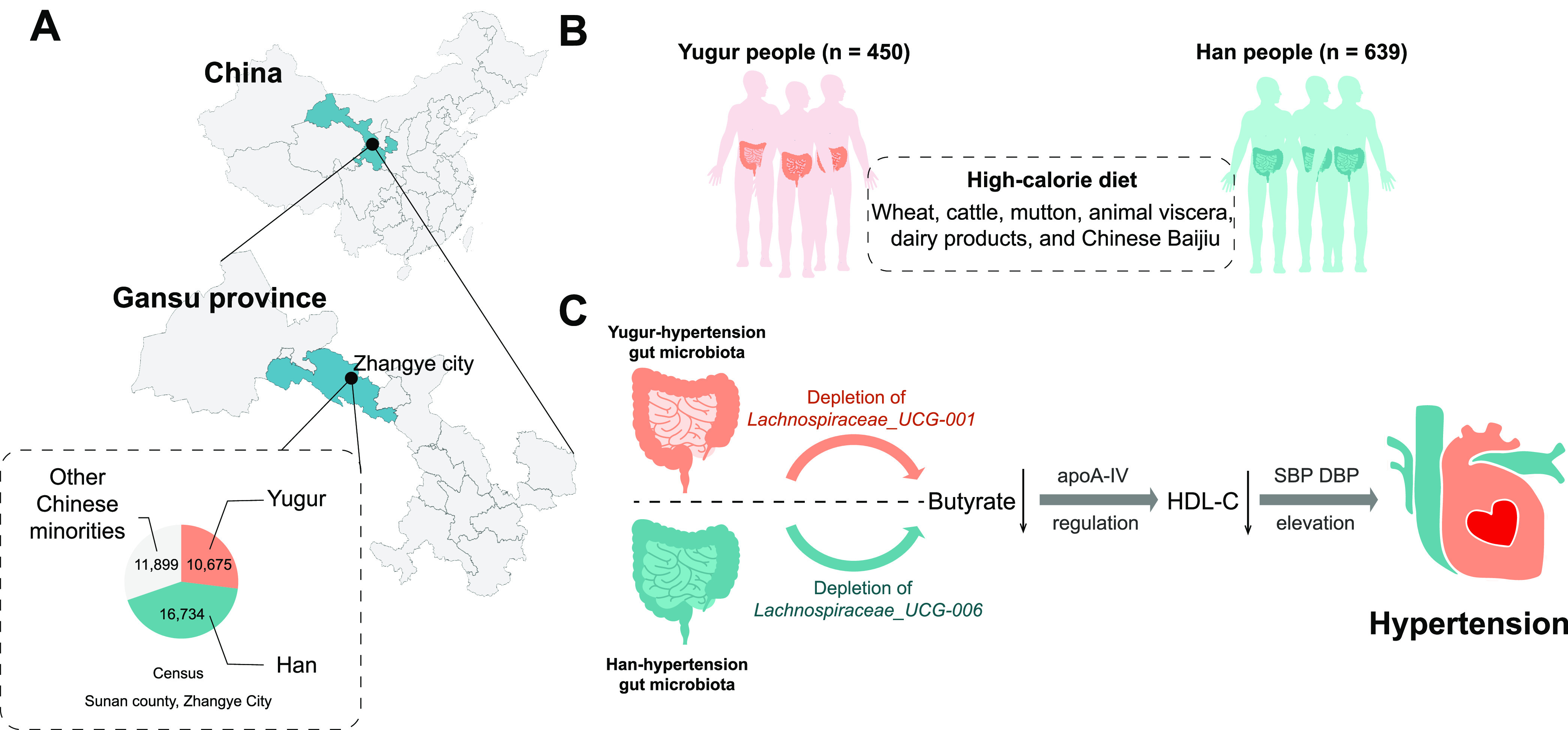
The recruited cohort and potential link between the gut microbiota and hypertension. (A) Members of the recruited cohort resided in Sunan County, Gansu Province, China. The local populational proportions are shown in the pie chart. (B) Yugur people (*n* = 450) and Han people (*n* = 639) who had been living in Sunan County for more than 15 years were investigated for the epidemic survey of hypertension prevalence and dietary customs in this study. (C) The potential ethnicity-specific mechanism proposed in this study, that the gut microbiota might promote hypertension pathogenesis by reducing intestinal butyrate and lowering HDL-C levels.

The Yugur, an Chinese ethnic group with a population of only 14,378, emerged around the eighth century by gathering mainly the Hexi Uighur and a few Mongolians, Tibetans, and people of other ethnicities. The Yugur reside in Sunan County, which is located in the middle of the Hexi Corridor, at the north foot of the Qilian Mountain in Northwest China, with a length of more than 650 km and an average altitude of 3,200 m (Fig. 1A). This area has an alpine semiarid climate with an annual average temperature of 4°C, and has low population mobility and a sparse population. Due to the unique natural environment, the Yugur have developed a special high-calorie diet based on wheat, cattle, mutton, animal offal, dairy products, and Chinese baijiu, with a limited intake of vegetables and fruits. Moreover, since the establishment of Sunan County in 1954, the Han who have successively immigrated to this area have been assimilated to Yugur customs, sharing a similar high-calorie diet. The high-calorie diet might be one of the causes of their high incidence of hypertension. According to our recent epidemiological survey of essential hypertension in Sunan County, the prevalence of essential hypertension among Yugurs was 43.3%, and that among Hans was 38.2%, both of which were higher than China’s national average (23.2% from 2012 to 2015) (12). The high-calorie diet may also equip Yugur and Han individuals with a distinct gut microbial composition, therefore influencing the pathogenesis of hypertension, but the gut microbial patterns and regulatory mechanisms behind this proposed modulating process remain unknown.

In this study, we investigated a total of 1,242 Yugur and Han people who had lived in Sunan County for more than 15 years and accounted for 3% of the local population to investigate the association and possible mechanism of the gut microbiota in the pathogenesis of hypertension under a high-calorie diet. Due to the limited population mobility and unique high-calorie dietary habits, this cohort could be representative for elucidating the associations between the gut microbiota and hypertension in the presence of a high-calorie diet.

## RESULTS

### Han and Yugur people in Sunan County present a high prevalence of hypertension with a high-calorie diet.

We conducted an epidemiological survey of essential hypertension and investigated the dietary structures for a total of 1,089 randomly selected individuals in Sunan County in 2015, including 639 Han people and 450 Yugur people (Fig. 1B; see also Table S1 in the supplemental material). The prevalence of hypertension was 38.2% in Han people, which was lower than that in Yugur people, at 43.3%. Both of these prevalences of hypertension were higher than China’s national average (23.2% from 2012 to 2015) (12). As for the dietary structure, we found that both Han and Yugur people shared a high-calorie diet according to dietary guidelines for Chinese residents (13) as a reference: (i) an excessive intake of meat (~178.8 to 234.9 g/day), which was more than the requirement of Chinese dietary guidelines (~50 to 100 g/day), and (ii) a limited intake of vegetables and fruits (~325.7 to 387.5 g/day), which was less than the requirement of Chinese dietary guidelines (~500 to 700 g/day). Furthermore, compared to people without hypertension, people with hypertension consumed more beef and mutton, animal offal, fried food, milk and its products, edible oil, and Chinese baijiu (*P < *0.05). These results have clarified a higher hypertension rate and a high-calorie diet in Han and Yugur people in Sunan County.

### The gut microbiota was dysbiotic in Han and Yugur people with hypertension.

For the microbial study, we collected and sequenced 153 fecal samples from Han and Yugur people who had been living in Sunan County for at least 15 years. This cohort consisted of 113 Han people (55 with hypertension and 58 without hypertension) and 40 Yugur people (23 with hypertension and 17 without hypertension). We balanced the numbers of samples with and without hypertension in this cohort for the following comparison analyses. There were no significant differences in sex, hypertension rate, age, or body mass index (BMI) between the recruited Han and Yugur people (*P > *0.05 [by a Mann-Whitney-Wilcoxon test]) (Table 1; Table S2). We found that Yugur people had a higher content of low-density lipoprotein cholesterol (LDL-C) and had a higher intake of coarse cereals, milk, and Chinese baijiu but a lower intake of rice and vegetables than Han people (*P < *0.05) (Table 1). In addition, we found that several dietary factors were correlated with their microbial compositions (false discovery rate [FDR] of <0.1 [by permutational multivariate analysis of variance {PERMANOVA}]) (Table S3), such as wheat, rice, coarse cereals, vegetables and fruits, animal offal, butter, and edible oil. Moreover, we compared the taxonomic abundances at the phylum, class, order, family, and genus levels between Han and Yugur people; no significant difference was found, except for the phylum *Firmicutes*, which was more enriched in Yugur people (FDR of <0.1) (Table S4). In addition, their overall gut microbial compositions cannot be separated in the principal-coordinate analysis (PCoA) (Fig. S1A).

**TABLE 1:** Demographic information of the cohort for the gut microbial study^*a*^

Characteristic	Value for microbiome cohort		*P*
	Han (*n* = 113)	Yugur (*n* = 40)	
No. of female subjects (%)	64 (56)	20 (50)	5.89E−01
No. of subjects with hypertension (%)	58 (51)	23 (57)	6.26E−01
Mean age (yrs) (IQR)	54 (35)	55.5 (22.25)	8.11E−01
Mean BMI (IQR)	24.51 (5.35)	25.39 (4.95)	1.34E−01
Mean blood pressure (IQR)			
SBP (mm Hg)	140 (26)	140.5 (23)	3.91E−01
DBP (mm Hg)	80 (18)	89 (22.5)	1.41E−01
FBG (mmol/L)	5.31 (0.547)	5.24 (0.66)	5.63E−01
PBG (mmol/L)	6.24 (0.425)	6.4 (0.45)	8.41E−02
Mean liver function (IQR)			
ALT (U/L)*	21 (15.22)	26 (15)	3.18E−02
TbIL (μmol/L)	12.6 (5.55)	12.2 (5.3)	1.56E−01
ALB (g/L)	44.65 (5.15)	45.9 (3.3)	2.32E−01
GLB (g/L)	27.75 (4)	28 (4.9)	9.74E−01
Mean renal function (IQR)			
BUA (mmol/L)	321.9 (117.2)	309.5 (147.9)	5.33E−01
CREA (μmol/L)*	65.2 (18.9)	58 (13.35)	1.04E−02
Mean blood fat content (mmol/L) (IQR)			
TCHO	4.12 (1.26)	4.59 (1.53)	1.75E−01
TAG	1.6 (1.2)	1.4 (1.02)	3.66E−01
LDL-C*	2.35 (1.26)	2.69 (1.22)	3.57E−02
HDL-C	1.22 (0.43)	1.23 (0.51)	6.09E−01
Mean dietary content (g/day) (IQR)			
Wheat and its products	341 (115)	345 (79.7)	9.52E−01
Rice and its products***	56 (22)	34.5 (18.25)	2.45E−11
Coarse cereals***	11 (7)	25.5 (12.25)	2.40E−11
Potato and its products	11 (5)	9 (6)	4.16E−01
Bean and its products**	22 (13)	18 (13.25)	5.94E−03
Vegetables and fruits**	376 (83)	330 (87.5)	1.45E−03
Beef and mutton	188 (67)	175 (93.5)	6.55E−01
Poultry meat*	29 (11)	26 (8.25)	3.44E−02
Animal offal***	14 (10)	20 (8.25)	3.04E−06
Fried food	15 (7)	15 (11)	8.35E−01
Milk and its products***	60 (17)	99.5 (35)	2.16E−13
Butter***	7 (5)	13 (10.5)	8.97E−06
Edible oil	51 (20)	50.5 (27.5)	3.02E−01
Chinese baijiu***	29 (22)	44.5 (34.5)	5.40E−06

a *P* values were calculated using the Mann-Whitney-Wilcoxon test (*, *P* < 0.05; **, *P < *0.01; ***, *P < *0.001). IQR, interquartile range; SBP, systolic blood pressure; DBP, diastolic blood pressure; FBG, fasting blood glucose; PBG, postprandial blood glucose; ALT, alanine aminotransferase; TbIL, total bilirubin; ALB, albumin; GLB, globulin; BUA, blood uric acid; CREA, creatinine; TCHO, total cholesterol; TAG, triacylglycerol; LDL-C, low-density lipoprotein cholesterol; HDL-C, high-density lipoprotein cholesterol.

**FIG 2:**
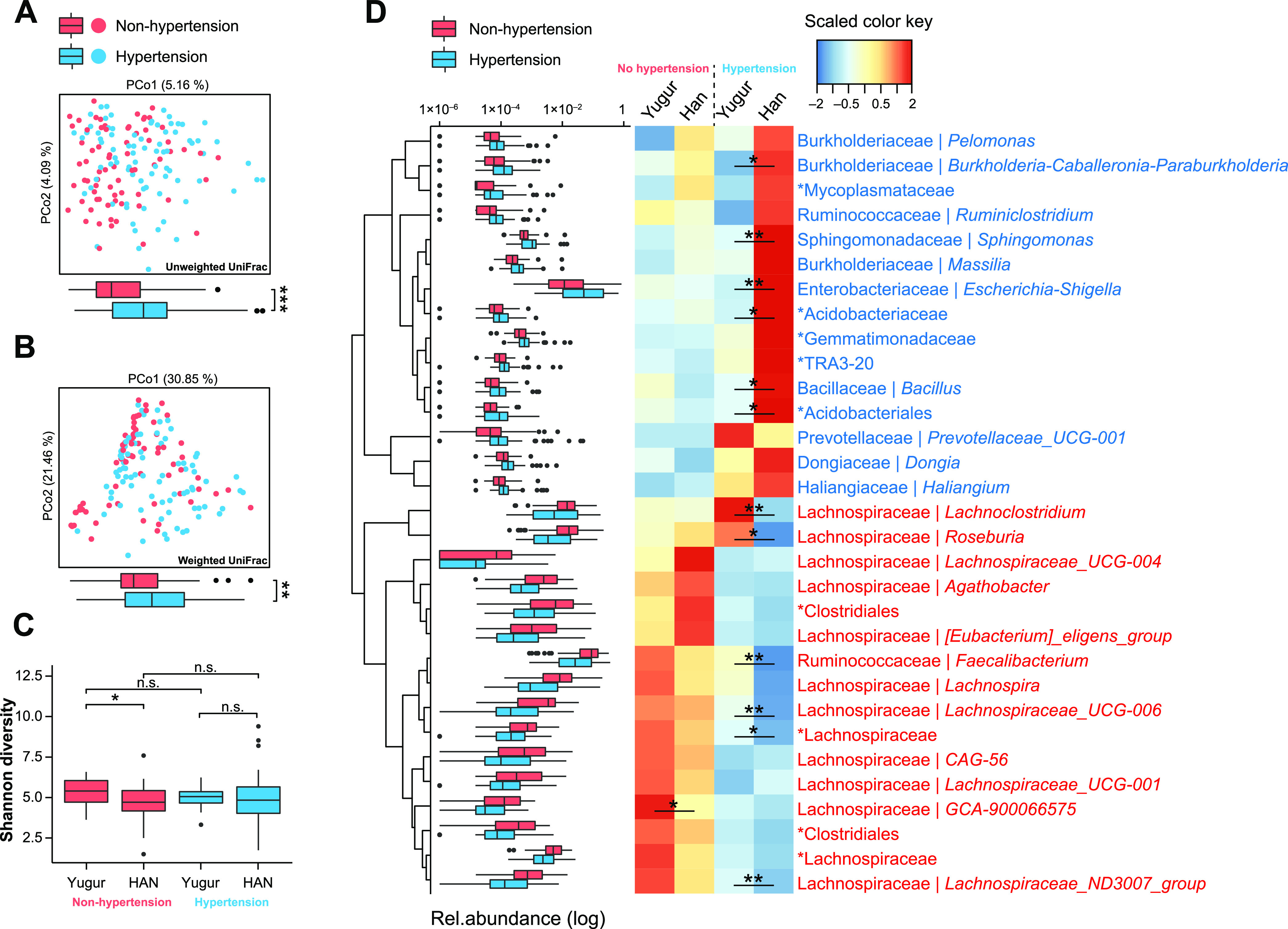
Differences in microbiota compositions between Han and Yugur individuals with and without hypertension. (A and B) Individual gut microbiota compositions of 81 hypertension patients and 72 nonhypertension individuals plotted on an unweighted UniFrac PCoA plot (A) and a weighted UniFrac PCoA plot (B), with the box plots below each one showing sample distributions. (C) Box plot showing the microbiota Shannon diversity of 17 Yugur without hypertension, 55 Han without hypertension, 23 Yugur with hypertension, and 58 Han with hypertension. (D, left) Box plots showing the relative abundances of 31 specific genera that had significantly different distributions between the hypertension and nonhypertension groups (*P < *0.05; *q* < 0.05 [by a Mann-Whitney-Wilcoxon test]). Hierarchical Ward linkage clustering was based on the Euclidean distance of the abundances of these genera among all the 153 samples. (Right) Heat map showing the scaled mean abundances of these genera in four subgroups as described above for panel C. Significance between subgroups is annotated in the heat map. The classified genera are annotated with the family and genus names, and the unclassified genera are designated with a higher rank with an asterisk. In all of the panels, statistical significance was tested using the Mann-Whitney-Wilcoxon test (*, *P < *0.05; **, *P < *0.01; n.s., not significant). The boxes represent the 25th to 75th percentiles, the black lines indicate the medians, and the whiskers extend to the maximum and minimum values within 1.5 times the interquartile range.

To explore differences in microbial compositions between hypertension and nonhypertension, we first performed PCoA on all of the fecal samples using unweighted (Fig. 2A) and weighted (Fig. 2B) UniFrac distances. We found that hypertension samples were evidently separated from nonhypertension samples against the PCo1 axis when both distances were used (*P* = 5.08 × 10^−5^ and *P* = 4.48 × 10^−3^ [by a Mann-Whitney-Wilcoxon test]). We also tested the differences between hypertension and nonhypertension groups in the PCoA for each of the ethnic groups separately (Fig. S1B and C) and found that the separation remained significant against the PCo1 axis in the Han group (*P *= 5.9 × 10^−5^) and the PCo2 axis in the Yugur group (*P* = 6.9 × 10^−3^). In addition, Yugur people without hypertension had higher microbial Shannon diversity than did Han people (*P* = 0.016), although microbial Shannon diversity showed no significant differences between hypertension and nonhypertension in both ethnic groups (Fig. 2C).

We then identified a total of 5 microbial phyla, 8 classes, 23 orders, 36 families, and 54 genera that were significantly elevated or depleted (*P < *0.05; *q* < 0.1 [by a Mann-Whitney-Wilcoxon test]) in the gut microbiota of people with hypertension (called the hypertension microbiota) compared to that of people without hypertension (called the nonhypertension microbiota) (Fig. 2D; Table S5). Among these 54 genera, 31 genera with *q* values of <0.05 were designated hypertension-related genera. The levels of a total of 15 hypertension-related genera were found to be significantly elevated in the hypertension microbiota, such as *Ruminiclostridium* (*P *= 4.56 × 10^−3^; *q* = 0.036), whose metabolic pathways were related to blood pressure regulation (14), and Escherichia*-Shigella* (*P *= 3.20 × 10^−4^; *q* = 4.14 × 10^−3^), whose infection in gastroenteritis was correlated with an increased risk of hypertension (15). Moreover, we observed elevations in the levels of *Pelomonas* (*P *= 1.63 × 10^−3^; *q* = 0.015) and *Sphingomonas* (*P *= 1.86 × 10^−4^; *q* = 0.038), which have been reported to be found in the blood microbiome and positively correlated with a few inflammatory markers (16) and the risk of hypertension (17), respectively. It was speculated that these two gut microbes might transit to the blood microbiome to promote hypertension, under circumstances of increased gut permeability in people with hypertension (18), which deserves further investigations.

### Depletion of *Lachnospiraceae* genera dominates microbial dysbiosis in Han and Yugur people with hypertension.

Notably, a total of 16 hypertension-related genera, significantly depleted in the hypertension microbiota, were found mostly from the family *Lachnospiraceae* (Fig. 2D; Table S5), such as *Lachnospiraceae* UCG-001 (*P *= 6.50 × 10^−3^; *q* = 0.046), *Lachnospiraceae* UCG-004 (*P *= 1.48 × 10^−4^; *q* = 3.77 × 10^−3^), *Lachnospiraceae* UCG-006 (*P* = 9.37 × 10^−8^; *q* = 2.06 × 10^−5^), *Lachnospira* (*P* = 1.18 × 10^−5^; *q* = 8.67 × 10^−4^), *Agathobacter* (*P* = 2.74 × 10^−5^; *q* = 1.50 × 10^−3^), *Faecalibacterium* (*P *= 1.41 × 10^−4^; *q* = 3.77 × 10^−3^), and *Roseburia* (*P* = 2.18 × 10^−4^; *q* = 4.00 × 10^−3^). Gut microbes belonging to the family *Lachnospiraceae* were reported to impact human hosts by producing short-chain fatty acids, converting primary to secondary bile acids (19–21), and facilitating colonization resistance against intestinal pathogens (22, 23). *Roseburia* species, for instance, have been reported to protect against atherosclerosis by generating butyrate (24). These results implied an important role of *Lachnospiraceae* genera in the pathogenesis of hypertension in Han and Yugur people.

### Yugur people with hypertension presented less altered microbiota.

We noticed that among the 31 hypertension-related genera identified in our study, only a significant elevation of *Haliangium* (*P *= 0.042) and only significant depletions of *Lachnospiraceae* UCG-001 (*P* = 4.43 × 10^−3^), GCA-900066575 (*P *= 3.31 × 10^−3^), and two unclassified genera, one of the family *Lachnospiraceae* (*P *= 0.034) and one of the order *Clostridiales* (*P *= 0.032), were observed in the hypertension microbiota compared to the nonhypertension microbiota when investigating only Yugur people (Table S6). Nevertheless, except for *Lachnospiraceae* UCG-001 (*P* = 0.18), all of the 30 other genera showed significant elevations or depletions when investigating only Han people (Table S6). To test whether this resulted from the bias of the sample sizes of the two groups, we randomly selected 40 Han samples (22 with hypertension and 18 without hypertension) to test microbial differences. The results showed that 25 out of 31 of these hypertension-related genera remained significantly different between Han people with and those without hypertension (*P < *0.05) (Table S7).

Moreover, a certain number of genera in the Yugur hypertension microbiota were found to be less altered than those in the Han hypertension microbiota (Fig. 2D). Compared to the Han hypertension microbiota, the levels of *Burkholderia-Caballeronia-Paraburkholderia* (*P *= 0.017), *Sphingomonas* (*P *= 8.97 × 10^−3^), Escherichia*-Shigella* (*P *= 2.51 × 10^−3^), *Bacillus* (*P *= 0.021), and two unclassified genera of the order *Acidobacteriales* (*P *= 0.036 and *P *= 0.018) were less elevated in the Yugur hypertension microbiota. In addition, *Lachnoclostridium* (*P *= 2.19 × 10^−3^) *Roseburia* (*P *= 0.032), *Faecalibacterium* (*P *= 5.08 × 10^−3^), *Lachnospiraceae* UCG-006 (*P *= 3.09 × 10^−3^), the *Lachnospiraceae* ND3007 group (*P *= 4.04 × 10^−3^), and an unclassified genus of the family *Lachnospiraceae* (*P *= 0.035) were less depleted in the Yugur hypertension microbiota. These results remained significant when using a subset of 40 Han samples (Table S7). These results suggested that Yugur people with hypertension had less altered microbiota, although the statistical significance might be biased by the different cohort sizes.

### The most discriminant microbial features of Han and Yugur people with hypertension.

**FIG 3:**
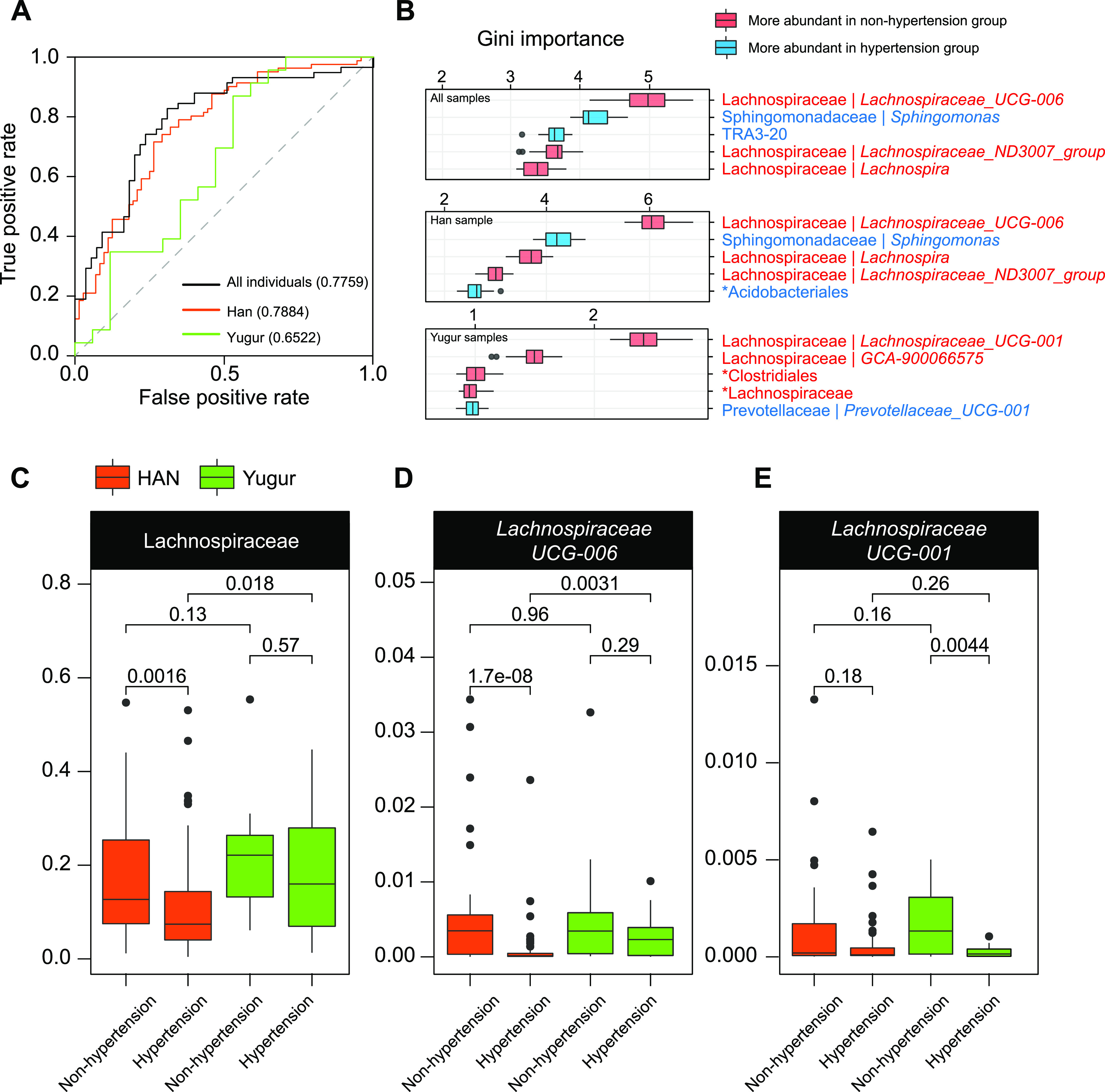
Microbial biomarkers for discriminating hypertension from nonhypertension. (A and B) The random-forest algorithm with 10 randomized 10-fold cross-validations was performed on 31 hypertension-related genera identified in Fig. 2, using all samples (*n* = 153), Han samples (*n* = 113), and Yugur samples (*n* = 40), to calculate the area under the receiver operating characteristic curve (AUROC) (A) and the Gini importance of each genus feature (B). The top five features are displayed and colored to show the group in which they are more significantly abundant (*P < *0.05; *q* < 0.05 [by a Mann-Whitney-Wilcoxon test]). (C–E) Box plots of the abundances of the most discriminant genus features and their family among hypertension and nonhypertension groups of Han and Yugur. Statistical significance was calculated by a Mann-Whitney-Wilcoxon test. The boxes represent the interquartile ranges between the first and third quartiles, and the lines inside represent the medians. Whiskers denote the lowest and highest values within 1.5 times the interquartile range from the first and third quartiles, respectively.

To explore the most discriminant microbes between the hypertension and nonhypertension groups, we then performed the random-forest algorithm on the whole cohort (*n* = 153), Han people (*n* = 113), and Yugur people (*n* = 40) (Fig. 3A). The Han hypertension microbiota could be discriminated from the Han nonhypertension microbiota with the best area under the receiver operating characteristic curve (AUROC) (0.7884), while the AUROC values were 0.7759 for all of the samples and only 0.6522 when applied to Yugur people. We also applied the random-forest algorithm to the subset of 40 Han samples and their combination with all 40 Yugur samples (Fig. S1D). The results turned out to be the same: the AUROC values were 0.8005 for the Han subset and 0.7695 for the combination. *Lachnospiraceae* genera were the most discriminant features for each ethnic group to discriminate hypertension microbiota from nonhypertension microbiota (Fig. 3B; Tables S8 to S10), with *Lachnospiraceae* UCG-006 for the Han group and *Lachnospiraceae* UCG-001 for the Yugur group. Moreover, we found that the family *Lachnospiraceae* was largely depleted in the Han hypertension microbiota (*P *= 1.6 × 10^−3^), while it was maintained at the same level in the Yugur hypertension microbiota as that in the nonhypertension microbiota (Fig. 3C). Moreover, *Lachnospiraceae* UCG-006 was notably depleted in the Han hypertension microbiota (*P *= 1.7 × 10^−8^) (Fig. 3D) but not in the Yugur hypertension microbiota. On the contrary, *Lachnospiraceae* UCG-001 was significantly depleted in the Yugur hypertension microbiota (*P *= 4.4 × 10^−3^) (Fig. 3E) but not in the Han hypertension microbiota.

### Depletion of *Lachnospiracea*e genera might promote hypertension by lowering the serum level of HDL-C.

**FIG 4:**
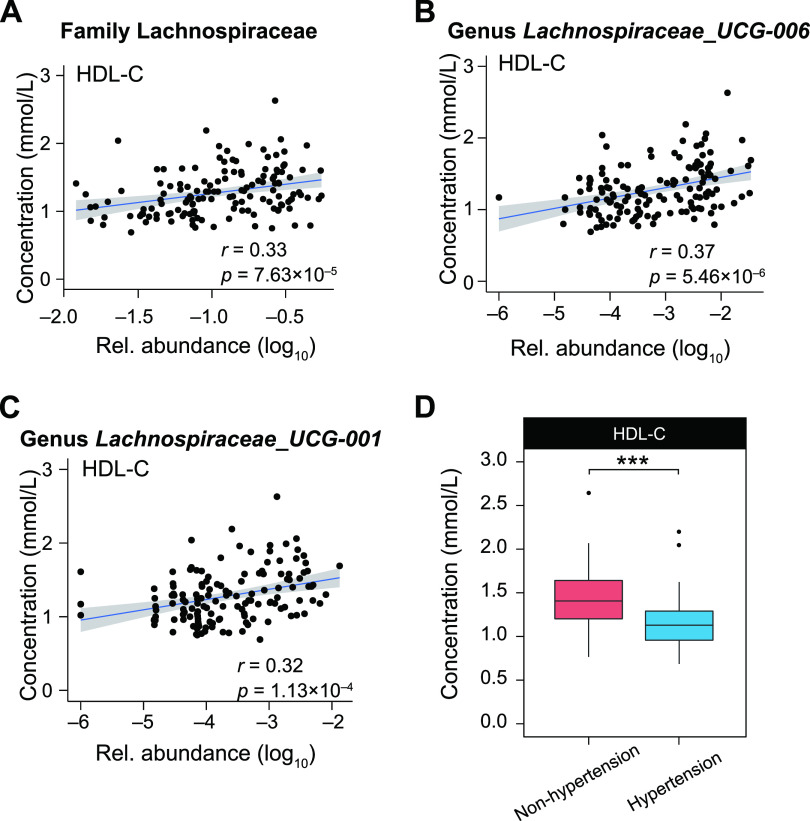
Correlations of *Lachnospiraceae* members with high-density lipoprotein cholesterol. (A to C) Scatterplots of the concentration of log_10_-transformed relative abundances of gut microbes (*x* axis) and high-density lipoprotein cholesterol (HDL-C) (*y* axis). The blue line is plotted using linear regression, with the 95% pointwise confidence interval band shaded gray. The correlation and statistical significance were calculated using Spearman correlation analysis. (D) Box plots showing the differences in concentrations of HDL-C between the hypertension group and the nonhypertension group. Statistical significance was calculated by a Mann-Whitney-Wilcoxon test (***, *P < *0.001). The boxes represent the interquartile ranges between the first and third quartiles, and the lines inside represent the medians. Whiskers denote the lowest and highest values within 1.5 times the interquartile range from the first and third quartiles, respectively.

We subsequently explored the correlations of the recognized hypertension-related microbes with the physiological properties of people (Fig. 4). Seven physiological properties were found to be significantly changed in people with hypertension compared to people without hypertension (*P < *0.05; *q* < 0.1) (Table S11). We then performed Spearman correlation analysis on these seven physiological properties with 31 hypertension-related genera and 20 hypertension-related families (Tables S5 S12). Seven properties were significantly correlated with 48 microbial taxa (*P < *0.05; *q* < 0.05 [by Spearman correlation analysis]) (Table S12). Systolic blood pressure (SBP) was positively correlated with the families *Desulfovibrionaceae* (*r* = 0.28; *P *= 4.04 × 10^−4^; *q* = 0.013) and *Mycoplasmataceae* (*r* = 0.27; *P *= 7.79 × 10^−4^; *q* = 0.013) and the genus Escherichia*-Shigella* (*r* = 0.25; *P *= 2.13 × 10^−3^; *q* = 0.011), while it was negatively correlated with the family *Lachnospiraceae* (*r* = −0.21; *P *= 8.29 × 10^−3^; *q* = 0.04) and the genera *Lachnospiraceae* UCG-006 (*r* = −0.38; *P *= 8.76 × 10^−7^; *q* = 1.9 × 10^−4^) and *Lachnospiraceae* ND3007 group (*r* = −0.33; *P *= 4.07 × 10^−5^; *q* = 1.35 × 10^−3^). Diastolic blood pressure (DBP) was negatively correlated with the family *Lachnospiraceae* (*r* = −0.23; *P *= 4.96 × 10^−3^; *q* = 0.029) and the genera *Lachnospiraceae* UCG-006 (*r* = −0.35; *P *= 7.63 × 10^−6^; *q* = 5.52 × 10^−4^), *Lachnospiraceae* ND3007 group (*r* = −0.32; *P *= 4.36 × 10^−5^; *q* = 1.35 × 10^−3^), and *Lachnospira* (*r* = −0.32; *P *= 5.49 × 10^−5^; *q* = 1.35 × 10^−3^). These results suggested that the depletion of *Lachnospiraceae* genera might be related to the increase in blood pressure.

Next, we focused on two genera of the family *Lachnospiraceae*, *Lachnospiraceae* UCG-006 and *Lachnospiraceae* UCG-001, whose depletions were the most prominent changes in the Han and Yugur hypertension microbiota, respectively. The family *Lachnospiraceae* (*r* = 0.33; *P *= 5.12 × 10^−5^; *q* = 3.58 × 10^−3^) (Fig. 4A) and both the genera *Lachnospiraceae* UCG-006 (*r* = 0.37; *P *= 5.73 × 10^−6^; *q* = 5.51 × 10^−4^) (Fig. 4B) and *Lachnospiraceae* UCG-001 (*r* = 0.33; *P *= 7.68 × 10^−5^; *q* = 1.35 × 10^−3^) (Fig. 4C) were found to be positively correlated with the concentration of high-density lipoprotein cholesterol (HDL-C). Moreover, the HDL-C level in our data was found to be significantly lower in people with hypertension (Fig. 4D) and negatively correlated with SBP (*r* = −0.37; *P *= 6.49 × 10^−6^) and DBP (*r* = −0.31; *P *= 1.37 × 10^−4^), which was consistent with a previous study of 4,552 individuals in a South Korean cohort (25).

Furthermore, it has been reported that butyrate can stimulate the secretion of apolipoprotein A-IV (ApoA-IV), a lipid-binding protein, which modulated reverse cholesterol transport to increase serum HDL-C (26). We then randomly selected 17 individuals to test the content of serum butyrate. We found that both Han and Yugur people with hypertension had lower contents of butyrate than did people without hypertension: 67.21 ± 4.23 ng/mL in four Han individuals with hypertension versus 81.66 ± 3.06 ng/mL in four Han individuals without hypertension (*P *= 1.99 × 10^−3^ [by a Mann-Whitney-Wilcoxon test]) and 60.88 ± 8.25 ng/mL in four Yugur individuals with hypertension versus 76.21 ± 1.76 ng/mL in five Yugur individuals without hypertension (*P *= 0.031).

## DISCUSSION

In this study, we found that people with hypertension and a high-calorie diet exhibited gut microbial dysbiosis, represented by the considerable depletion of *Lachnospiraceae* genera. Moreover, we found that the depletion of *Lachnospiraceae* was correlated with a decrease in HDL-C and increases in SBP and DBP. Furthermore, we validated that the Han people and Yugur people with hypertension had lower serum butyrate contents. We concluded that the depletion of *Lachnospiraceae* genera could lead to decreased intestinal butyrate production in hypertensive individuals, which in turn contributed to a lower level of HDL-C via ApoA-IV gene regulation and led to an increase in blood pressure and the promotion of hypertension (Fig. 1C).

A diet rich in fruits, vegetables, and low-fat dairy products with reduced saturated and total fat has been recommended for people to ameliorate hypertension (8). However, the members of the recruited cohort in our study shared nearly opposite dietary customs, with an excessive intake of meat but a limited intake of vegetables or fruits, which may be a potential cause of their higher incidence of hypertension. Due to a paucity of vegetables in the diet, a stronger fermentation ability was needed to cope with the limited intake of plant polysaccharides. Additionally, the family *Lachnospiraceae* can ferment diverse plant polysaccharides to SCFAs (19, 21, 22, 27), which played a role in the maintenance of health, such as energy supply and immunity regulation (28, 29). Nonetheless, we found that the bulk of the considerably depleted microbes in the hypertension group came from the family *Lachnospiraceae*, which might weaken the host’s ability to ferment plant polysaccharides. The overall abundance of the family *Lachnospiraceae* was not significantly decreased in the Yugur hypertension microbiota, although it was considerably decreased in the Han hypertension microbiota. It was speculated that when hypertension developed, the Han gut microbiota might be more vulnerable than the Yugur gut microbiota, possibly due to the shorter time of their residence in Sunan County or the host genetic distinction. However, this speculation was limited by the different sample sizes in this study, which requires further investigation. Moreover, although the family *Lachnospiraceae* remained abundant in the Yugur hypertension microbiota, the genus *Lachnospiraceae* UCG-001 was significantly depleted in the Yugur hypertension microbiota but not in the Han hypertension microbiota. On the contrary, another genus, *Lachnospiraceae* UCG-006, was considerably reduced in the Han hypertension microbiota but not in the Yugur hypertension microbiota. Thus, the gut microbiota of the two ethnic groups might respond differently to the stress of hypertension, which might partly explain the disparity in hypertension prevalences in these two ethnic groups in the community.

Intestinal butyrate, accounting for 95% of the SCFAs produced by the gut microbiota (30), was reported to increase serum HDL-C by stimulating ApoA-IV gene expression (26). In addition, HDL-C was reported to be negatively correlated with SBP and DBP in a cohort of 4,552 South Koreans (25), which was consistent with our findings. Moreover, HDL-C also played an important role in reducing the risk of a variety of cardiovascular diseases (31). Therefore, HDL-C might be a crucial link between microbes and hypertension. In this study, we propose a potential mechanism, that the depletion of members of the family *Lachnospiraceae* caused less production of intestinal butyrate in people with hypertension (19, 21, 22, 27), which might contribute to the lower level of HDL-C, elevated SBP and DBP, and hypertension development. This mechanism would be significant for people who follow a high-calorie diet with a limited intake of vegetables. Besides the link between microbes, HDL-C, and blood pressure, we also found a certain number of direct correlations of microbes with SBP and DBP, such as the positive correlation of SBP with Escherichia*-Shigella*. Additionally, several increased intestinal microbes such as *Sphingomonas* were also reported to be increased in the blood of people with hypertension. Hence, further investigations are required to explore the multiple potential mechanisms in this study, such as the microbe-metabolite (butyrate and HDL-C)-SBP/DBP-hypertension link, the microbe-SBP/DBP-hypertension link, and the intestine-blood-microbe-hypertension link.

This study also has limitations. First, owing to the lack of dietary information for the Chinese reference population, we cannot give a conclusion about whether the high-calorie diet can actually increase the risk of hypertension. In this study, the high-calorie diet served only as a background for the microbial study. Second, since the different cohort sizes of Han and Yugur people could influence the statistical significance of microbial alterations, we may not be able to give a strong conclusion about the ethnic differences in microbial dysbiosis. Nevertheless, we randomly selected a subset of 40 Han samples for the comparison analysis to validate these observed results. We found that most of the results remained significant when using the subset of Han samples. Third, we tested the serum butyrate contents of only some of the individuals in this study owing to strict policies for blood testing in Sunan County. However, all of these limitations would not negate the substantial microbial differentiation between people with and those without hypertension under a high-calorie diet as well as the strong correlations between dysbiosis and HDL-C, which was of clinical importance. Moreover, butyrate might be one of the potential media through which microbes adjusted HDL-C, and further research into the underlying mechanisms is necessary.

### Conclusions.

This study demonstrates that individuals with hypertension under a high-calorie diet exhibit a substantial depletion of *Lachnospiraceae* genera, which might promote hypertension progression by lowering serum HDL-C levels. This study provides new insight into the link between microbial dysbiosis and hypertension under a high-calorie diet. Further investigations of the role of the gut microbiota in HDL-C regulation in a variety of cardiovascular diseases are warranted.

## MATERIALS AND METHODS

### Ethical statement.

All procedures performed in this study were approved by the Medical Ethics Committee of Northwest Minzu University (no. XBMZ-YX-202004) and were performed in accordance with the Declaration of Helsinki of 1975. All participants provided written informed consent to take part in the study.

### Cohorts for the epidemiological survey and gut microbial study.

A cohort of 1,089 Han (*n* = 639) and Yugur (*n* = 450) individuals in Sunan County was randomly recruited for the epidemiological survey in 2015. This cohort was used mainly to support the background of this study, that Han and Yugur in Sunan had high hypertension rates and had a special high-calorie diet. Another cohort of 153 Han (*n* = 113) and Yugur (*n* = 40) individuals was recruited for the gut microbial study in 2020. For both cohorts, hypertension was defined as a persistent elevation of systolic blood pressure (SBP) of ≥140 mm Hg and/or diastolic blood pressure (DBP) of ≥90 mm Hg. Information on dietary intake was obtained using a food frequency questionnaire. The average intake of specific foods was calculated according to the intake of foods per meal and the frequency of foods during a month. To rationally remove the effects of other environmental confounders unrelated to this study, all of the recruited individuals in this study were aged >18 years and had been living in Sunan County for more than 15 years. For the cohort of the microbial study, demographic data such as sex, hypertension rate, age, BMI, blood pressure, blood parameters, and diet were compared (Table 1).

### Fecal sample collection.

A total of 153 fecal samples from Yugur and Han people were collected for the gut microbial study. A total of 10 g of feces from each sample was collected into a stool storage tube containing stool preservation fluid in the morning. The preservation fluid and stool sample were mixed evenly before the sample was frozen in a −80°C freezer for ≥24 h. Within 1 week, we shipped samples on dry ice to the laboratory for the following experiments. All of the participants must not have taken any antibiotics, microbial preparations, or antidiarrheal or weight loss drugs and must not have had a history of diarrhea or other gastrointestinal (GI) diseases within the last month. The dietary information for these 153 individuals was collected.

### DNA extraction and 16S rRNA gene sequencing.

A PowerSoil DNA extraction kit (Qiagen, Hilden, Germany) was used to extract genomic DNA from fecal samples according to the manufacturer’s instructions. We used 1% agarose gel electrophoresis and a NanoDrop2000 spectrophotometer (Thermo Fisher Scientific, Waltham, MA, USA) to measure DNA concentration and purity, respectively. A suitable amount of the sample was added to a centrifuge tube, and sterile water was used to dilute the sample to 1 ng/μL. We used the diluted DNA as a template and specific primers 343F (5′-TACGGRAGGCAGCAG-3′) and 798R (5′-AGGGTATCTAATCCT-3′) with Tks Gflex DNA polymerase for PCR amplification of the 16S V3-V4 region in samples to ensure amplification efficiency and accuracy. The first round of PCR amplification conditions consisted of predenaturation at 94°C for 5 min followed by 26 cycles of 94°C for 30 s, 56°C for 30 s, and 72°C for 20 s and then a final extension step at 72°C for 5 min with holding at 4°C. The second round consisted of predenaturation at 94°C for 5 min followed by seven cycles of 94°C for 30 s, 56°C for 30 s, and 72°C for 20 s and then a final extension step at 72°C for 5 min with holding at 4°C. Illumina (San Diego, CA, USA) MiSeq sequencing was used to generate paired-end (PE) sequences.

### Sequence processing.

Trimmomatic software version 0.35 was used to remove sequences with moving windows whose mean base quality was <20 and sequences that were <50 bp long (32). Fast Length Adjustment of Short Reads (FLASh) software version 1.2.11 was used to join PE sequences after removing impurities (33). The parameters used for joining were as follows: a minimum overlap of 10 bp, a maximum overlap of 200 bp, and a maximum rate of 20%. Quantitative Insights into Microbial Ecology (QIIME) split_libraries.py software version 1.8.0 was used to remove PE sequences containing N bases and sequences with a base quality score (*Q*_20_) of <75% (34). UCHIME software version 2.4.2 was used to remove chimeras from the remaining sequences (35). *De novo* operational taxonomic unit (OTU) picking was performed: Vsearch software version 2.4.2 was used for OTU clustering with 97% similarity (36), and the sequence with the highest abundance in each OTU was taken as the representative sequence for the RDP classifier (37). A naive Bayesian classification algorithm was used to align and annotate representative sequences. Rarefaction was set at 35,490 reads based on the curve plateaus for alpha diversity.

### Principal-coordinate analysis.

Unweighted and weighted UniFrac distances between OTUs among all samples were used for principal-coordinate analysis (PCoA). The R function dudi.pco in the R package ade4 was used to perform PCoA, and the R package ggplot2 was used to visualize the results.

### Identification of microbial biomarkers.

The random-forest algorithm was used to identify the microbial biomarkers for each of the groups. Ten randomized 10-fold cross-validations were performed on microbial features to calculate the mean values of the decrease of the Gini score as the feature importance. The area under the receiver operating characteristic curve (AUROC) was calculated by performing the random-forest algorithm on all samples, Yugur samples, and Han samples.

### Quantitative detection of serum butyrate.

Eighty microliters of ice-cold acetonitrile-water (1:1 [vol/vol] containing [^2^H_9_]pentanoic acid and [^2^H_11_]hexanoic acid) was added to the 80-mg freeze-dried serum samples. Samples were extracted by ultrasonication for 10 min in an ice-water bath. Samples were then centrifuged at 4°C (12,000 rpm) for 10 min. For derivatization, 80 μL of the standard solution or 80 μL of the supernatants was mixed with 40 μL of 200 mM 3-nitrophenylhydrazine (NPH) in 50% aqueous acetonitrile and 40 μL of a 120 mM N-(3-dimethylaminopropyl)-N0-ethylcarbodiimide (EDC)–6% pyridine solution in the same solvent. The mixture reacted at 40°C for 30 min. Afterward, the samples were placed on ice for 1 min and then filtered through a 0.22-μm organic-phase pinhole filter for subsequent ultraperformance liquid chromatography-tandem mass spectrometry (UPLC-MS/MS) analysis. A pooled sample for quality control was prepared by mixing aliquots of all of the samples. The mixed standard stock solution was prepared and diluted to produce the calibration curve.

Liquid chromatography was performed on a Nexera LC-30A ultrahigh-performance liquid chromatography (UHPLC) system (Shimadzu). An Acquity UPLC BEH C_18_ column (100 by 2.1 mm, 1.7 μm) was used for analysis. The injection volume was 2 μL. Mobile phase A was water containing 0. 1% formic acid, and mobile phase B was acetonitrile. The following gradient elution procedure was used: 0 min A/B (90:10, vol/vol), 1 min A/B (90:10, vol/vol), 2 min A/B (75:25, vol/vol), 6 min A/B (65:35, vol/vol), 6.5 min A/B (5:95, vol/vol), 7.8 min A/B (5:95, vol/vol), 7.81 min A/B (90:10, vol/vol), and 8.5 min A/B (90:10, vol/vol). All of the samples were kept at 4°C during the analysis, and the column temperature was set at 40°C. Mass spectrometry was performed on the AB Sciex Selex Ion Triple Quad 5500 system. Sciex OS-MQ software was used for quantification. The concentration of butyrate was calculated according to the peak area and the calibration curve.

### Statistical analysis.

For the log_10_-transformed abundance of OTUs, we added a pseudocount of 0.000001 to those OTUs with an abundance of zero in a sample, as the minimum abundance. For categorical metadata, samples were pooled into bins (hypertension/nonhypertension, Yugur people/Han people, and Yugur hypertension/Han hypertension/Yugur nonhypertension/Han nonhypertension), and significance was calculated using the Mann-Whitney-Wilcoxon test (*P* values) with the Benjamini-Hochberg correction (FDR and *q* values) (38). To evaluate the effects of the bias for the sample sizes in this study, we randomly selected 40 Han samples from the 113 Han samples as a subset for the comparison analysis. The seed was set as 2,022 for the random-number generator in R. Permutational multivariate analysis of variance (PERMANOVA) was performed with 9,999 permutations using the unweighted UniFrac distance matrix. Age, gender, and body mass index were used as covariates. The significance of comparisons of serum butyrate contents was calculated using a *t* test. The correlations between variables were tested using Spearman correlation analysis.

### Data availability.

Sequencing data are available in the Genome Sequence Archive (GSA) of the National Genomics Data Center (project accession no. CRA005607).
