# Supplementary material for: High-Density Lipoprotein Cholesterol as a Potential Medium between Depletion of Lachnospiraceae Genera and Hypertension under a High-Calorie Diet
Source: Microbiol Spectr. 2022 Oct 17;10(6):e02349-22. doi: 10.1128/spectrum.02349-22 (PMC9769594; doi:10.1128/spectrum.02349-22)
Supplement: Supplemental file 1 — Fig. S1. Download spectrum.02349-22-s0001.pdf, PDF file, 0.1 MB [file spectrum.02349-22-s0001.pdf]

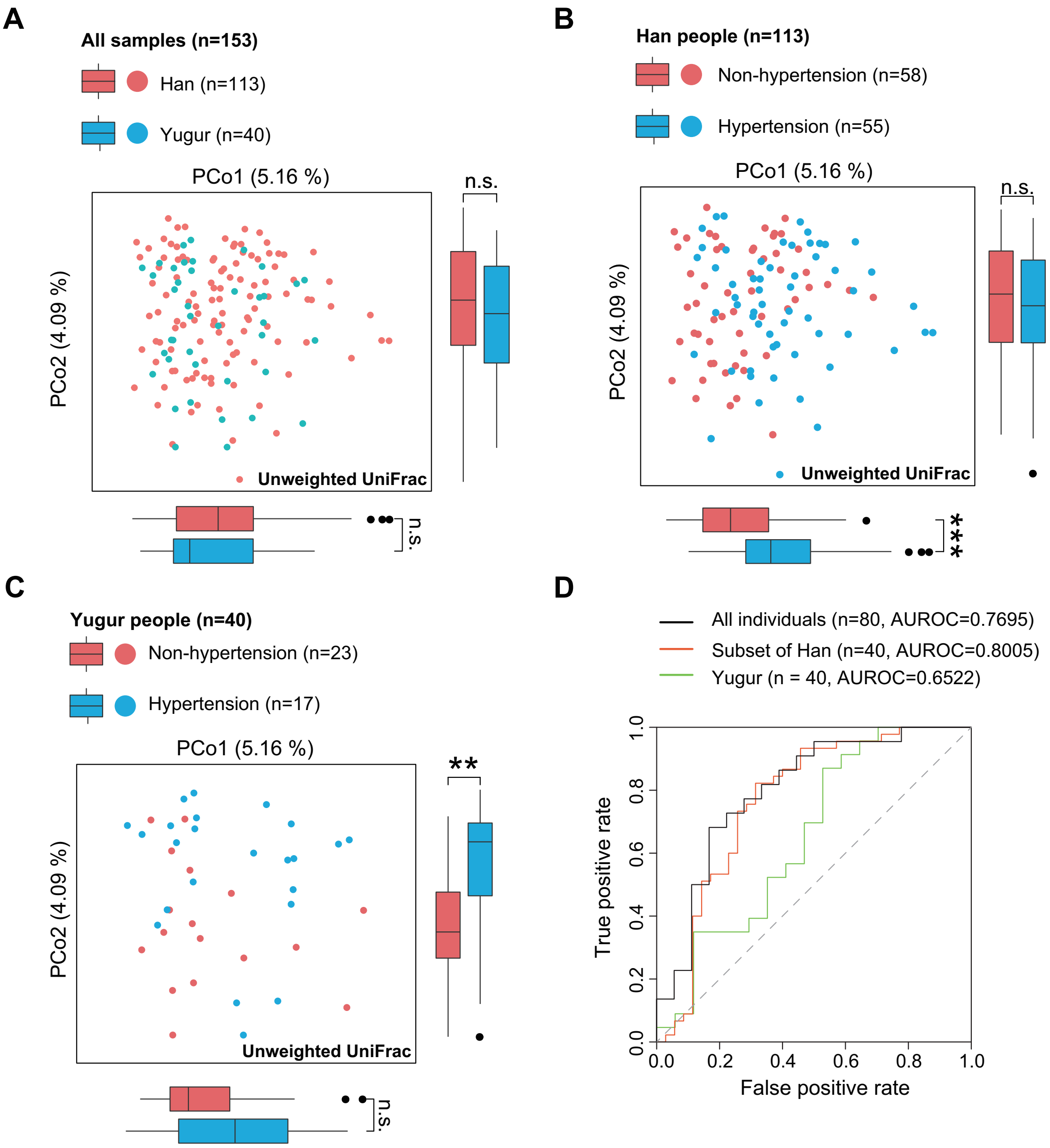

FIG S1 Gut microbial differentiation between groups. (A) Individual gut microbiota compositions of Han samples (n=113) and Yugur samples (n=40) plotted on an unweighted UniFrac PCoA plot. (B) Individual gut microbiota compositions of Han samples without hypertension (n=58) and with hypertension (n=55). (C) Individual gut microbiota compositions of Yugur samples without hypertension (n=23) and with hypertension (n=17). **, $P<0.01$; ***, $P<0.001$, n.s., not significant. Statistical significance was calculated by Mann-Whitney-Wilcoxon test. Boxes represent the interquartile range between first and third quartiles and the line inside represents the median. Whiskers denote the lowest and highest values within 1.5 × interquartile range from the first and third quartiles, respectively. (D) To remove the effects of sample size on the Randomforest results in Fig. 3A, a subset of 40 Han samples was used for analysis. A Randomforest algorithm with 10 randomized 10-fold cross validation was performed on 31 hypertension-related genera identified in Fig. 2, using combination of the subset of 40 Han samples, 40 Yugur samples, and their combination, to calculate Area Under the Receiver Operating Characteristic curve (AUROC), respectively
